# Therapeutic Effects of Sulforaphane on *Helicobacter pylori*-Infected Mice: Insights from High-Coverage Metabolomics and Lipidomics Analyses of Serum and Liver

**DOI:** 10.3390/ijms26167791

**Published:** 2025-08-12

**Authors:** Shuling He, Lvyun Sun, Jiali Chen, Yixin Li, Ying Pan, Amei Su, Qiuyao Mao, Jiaqian Hu, Disheng Feng, Yang Ouyang

**Affiliations:** 1 Department of Health Inspection and Quarantine, School of Public Health, Fujian Medical University, Fuzhou 350122, China; heshuling@fjmu.edu.cn (S.H.); sunly@fjmu.edu.cn (L.S.); 2230220251@fjum.edu.cn (J.C.); liyixin316@163.com (Y.L.); panying@stu.fjmu.edu.cn (Y.P.); 2562711829@fjmu.edu.cn (A.S.); mqy0167@fjmu.edu.cn (Q.M.); 3200333059@stu.fjmu.edu.cn (J.H.); 2 Fujian Key Laboratory of Chinese Materia Medica, Institute of Structural Pharmacology & TCM Chemical Biology, College of Pharmacy, Fujian University of Traditional Chinese Medicine, Fuzhou 350122, China

**Keywords:** sulforaphane, *Helicobacter pylori*, metabolomics, lipidomics

## Abstract

Sulforaphane, a natural isothiocyanate predominantly found in cruciferous vegetables, has shown potential in preventing and treating *Helicobacter pylori* infection. However, the underlying metabolic mechanisms remain largely unclear. This study employed high-coverage metabolomics and lipidomics methods to comprehensively investigate the effects of sulforaphane on the serum and liver metabolic profiles of *H. pylori*-infected mice. Metabolomics and lipidomics analysis revealed that *H. pylori* infection disrupted multiple metabolic pathways, leading to perturbations in amino acids, fatty acids, bile acids, and various lipid species. Sulforaphane treatment can ameliorate these disruptions, notably reversing alterations in serum glycerophospholipids and restoring hepatic levels of amino acids, bile acids, glycerophospholipids, ceramides, and peptides. Key metabolic pathways implicated included glutathione metabolism and glycine and serine metabolism, which are associated with antioxidant defense and host resistance to pathogenic infections. These findings offer a comprehensive metabolic basis for understanding the therapeutic effects of sulforaphane against *H. pylori* infection.

## 1. Introduction

*Helicobacter pylori* (*H. pylori*) is a microaerobic Gram-negative bacterium that colonizes the gastric mucosa. Approximately half of the global population is infected with this bacterium, and it is classified as a group 1 carcinogen by the World Health Organization [1]. *H. pylori* colonization in the gastric mucosa can induce a chronic immune response, leading to increased oxidative stress levels and subsequent damage to the gastric mucosa. This process can contribute to the development of various gastrointestinal diseases. Approximately 70% of gastric ulcers and up to 65–80% of gastric cancers are associated with *H. pylori* infection [1,2]. However, *H. pylori* infection does not typically present with obvious symptoms in the early stages, making it difficult to detect [3]. Therefore, daily dietary interventions may be a relatively effective method for its prevention and treatment.

Sulforaphane is abundantly present in cruciferous plants (e.g., cabbage, broccoli, or kale) as glucoraphanin. Myrosinase, an enzyme in these vegetables, hydrolyzes glucoraphanin to convert it into sulforaphane [4]. Sulforaphane exhibits antioxidant [5], antibacterial, and anticancer effects [6]. It can protect gastric mucosal cells from *H. pylori*-induced oxidative damage [7], inhibit and eradicate *H. pylori* [8], and reduce *H. pylori* colonization [9]. Despite these known benefits, the metabolic mechanisms underlying sulforaphane’s therapeutic effects against *H. pylori* infection remain largely unresolved. Therefore, further research is necessary to determine the potential of sulforaphane as a safe and effective dietary agent for managing *H. pylori* infections and to delineate its impact on host small molecular metabolite profiles.

Metabolomics is an analytical approach that enables the qualitative and quantitative analysis of metabolites, typically those with a relative molecular weight below 1500 Da. It is employed to study the dynamic metabolic responses to internal genetic mutations, pathophysiological changes, or external environmental stimuli [10]. Metabolomics can also identify potential biomarkers and facilitate in-depth investigation of disease development mechanisms and drug action, thereby laying a foundation for disease diagnosis, treatment, and the clinical application of drugs [11]. Lipidomics, a specialized branch of metabolomics, focuses on the comprehensive analysis of lipids, their interactions, and their biological roles. Integrating metabolomics and lipidomics provides extensive coverage of both polar metabolites and non-polar and polar lipids, offering a more comprehensive depiction of the metabolic landscape. This integrated approach enables robust network analysis, facilitating the identification of critical metabolic drivers in biological processes [12].

This study established an *H. pylori*-infected mouse model, to which sulforaphane was orally administered. Utilizing ultra-high-performance liquid chromatography–high resolution mass spectrometry (UHPLC-HRMS)-based non-targeted metabolomic and lipidomic approaches, we analyzed the metabolic profiles of serum and liver samples from these mice. The main objectives are to comprehensively analyze the metabolomic and lipidomic changes induced by *H. pylori* infection and to clarify the scope of sulforaphane’s repairing effects on the host metabolome and lipidome as well as the key metabolic pathways involved.

## 2. Results

### 2.1. Detection of H. pylori Colonization and Biological Characteristics of Mice

**Figure 1:**
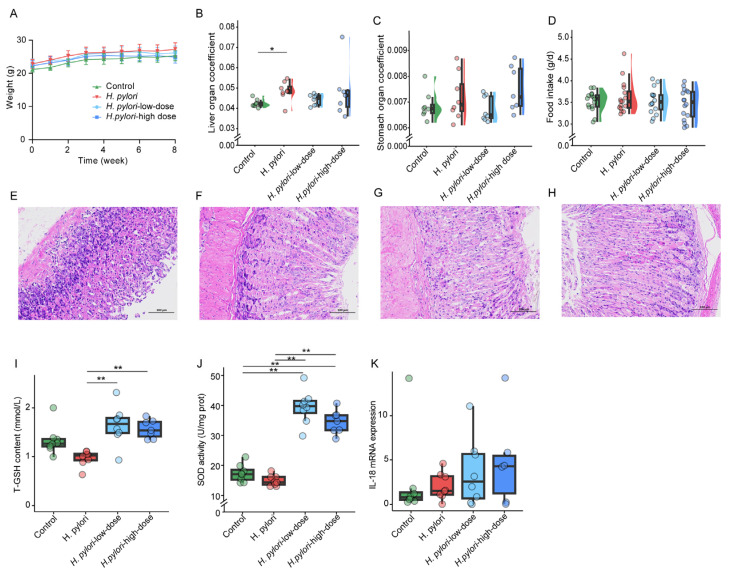
Biological characteristics of mice and H&E staining of mouse stomach sections. (**A**) The body weight curves of mice in each group. The (**B**) liver organ coefficient, (**C**) stomach organ coefficient, and (**D**) average food intake of mice in each group. Representative H&E-stained images of stomach section from (**E**) mouse in the control group, (**F**) *H. pylori*-infected mouse at 4 weeks post-infection, and *H. pylori*-infected mouse treated with (**G**) low-dose and (**H**) high-dose sulforaphane. Scale bars, 100 µm. The (**I**) T-GSH content, (**J**) SOD activity, and (**K**) IL-18 mRNA expression. “*” means *p* ≤ 0.05, and “**” means *p* ≤ 0.01. The *H. pylori*-low-dose and *H. pylori*-high-dose groups received 5 mg/kg/d and 20 mg/kg/d of sulforaphane, respectively.

To confirm the successful colonization of *H. pylori* in the stomach, one mouse was randomly selected from the *H. pylori* group after gavaging with *H. pylori* four times. The gastric mucosal tissue of the mouse was utilized for the detection of *H. pylori* colonization. The results are shown in Figure S1, where wells 1, 2, and 3 represent the sample test wells and well 4 served as the negative control. The samples tests results were positive, suggesting the successful colonization of *H. pylori* in the mouse stomach. Throughout the experiment, the body weights of mice in all groups exhibited an increasing trend, and there were no significant differences in body weights among the groups each week (Figure 1A). However, compared to the control group, the liver weight of mice infected with *H. pylori* significantly increased. The liver organ coefficient of the *H. pylori* group was also significantly higher than that of the control group. Notably, sulforaphane intervention was found to reduce the liver weight, resulting in a liver organ coefficient that was not significantly different from that of the control group (Figure 1B). Furthermore, the results of the one-way ANOVA indicated that there were no significant differences in the stomach organ coefficients among the groups of mice (Figure 1C). There were also no significant differences in the organ coefficients of the heart, lungs, kidneys, and spleen among the groups of mice (Figure S2). Subsequently, the food intake of the mice was calculated, and one-way ANOVA was performed. As shown in Figure 1D, there were no significant differences in the average food intake between the groups. The daily water intake of each mouse is shown in Figure S3. There is a significant difference in the average water intake between the control group mice and the other groups.

Pathological changes in the stomach tissues of mice were evaluated using H&E staining (Figure 1E–H). Stomach tissue analysis revealed degeneration of the epithelial cells in the gastric mucosa of *H. pylori*-infected mice. Morphologically, web-like vacuolar degeneration was observed, and some epithelial cells detached. Treatment with low-dose sulforaphane alleviated the vacuolar degeneration in the cytoplasm of surface epithelial cells, while high-dose sulforaphane demonstrated a more pronounced therapeutic effect.

As shown in Figure 1I,J, *H. pylori* infection led to a decline in both SOD and T-GSH levels in mouse gastric mucosa. Intervention with sulforaphane significantly upregulated the levels of both T-GSH and SOD, indicating a protective effect against oxidative stress induced by *H. pylori* infection. Conversely, the mRNA expression of IL-18 (Figure 1K) exhibits an upward trend post-*H. pylori* infection, with further elevation observed in the presence of sulforaphane intervention.

### 2.2. Data Quality Assessment of High-Coverage Metabolomics

**Figure 2:**
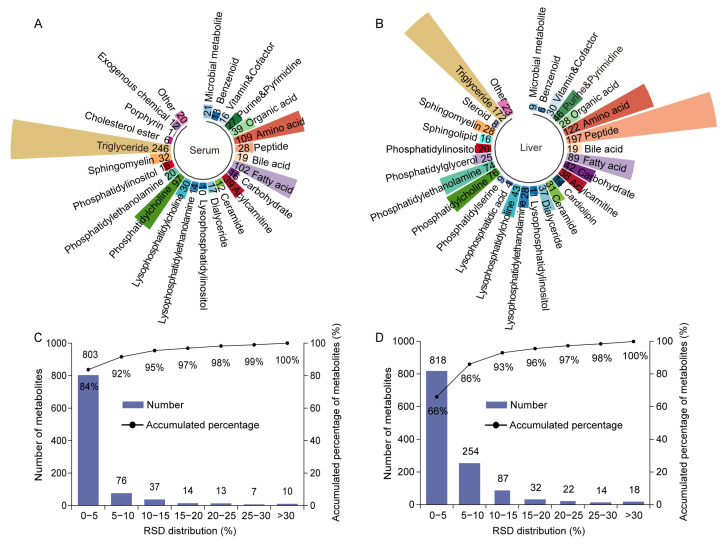
Detected metabolite classes and evaluation of metabolomics data quality. Circular bar chart of the detected classes and number of metabolites in mouse (**A**) serum and (**B**) liver. Distribution of relative standard deviations of metabolites in (**C**) serum QC samples and (**D**) liver QC samples.

To investigate the key metabolites and related metabolic pathways of sulforaphane in improving *H. pylori* infection, untargeted metabolomics analysis using UHPLC-HRMS was performed on mice serum and liver samples. A total of 960 metabolites belonging to 25 classes were detected in serum (Figure 2A), while 1245 metabolites belonging to 28 classes were detected in the liver (Figure 2B). As shown in Figure 2C, 99% of the metabolites in the serum QC samples had a relative standard deviation of less than 30%. The relative standard deviation of 98% of metabolites in liver QC samples was less than 30% (Figure 2D). These results indicate that the data are reliable and can be used for further analysis.

### 2.3. Integrated Metabolomics and Lipidomics Analysis of Mouse Serum

**Figure 3:**
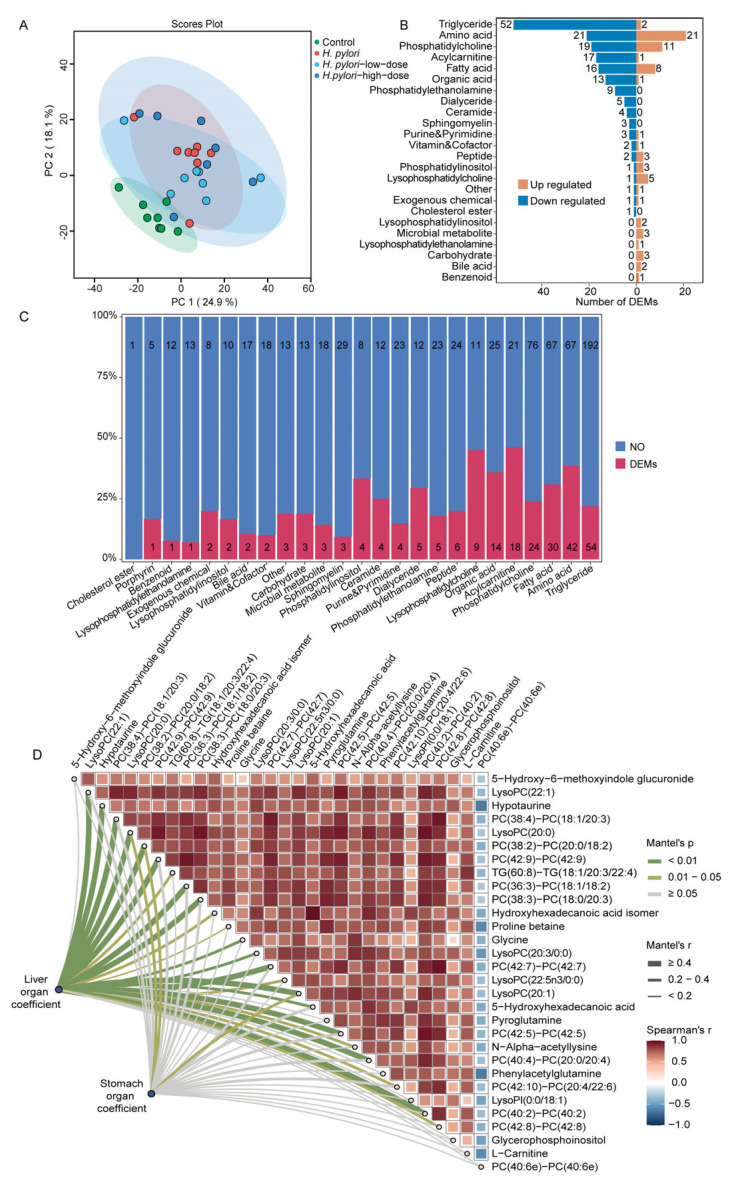
Metabolomics and lipidomics analysis of the effects of *H. pylori* infection on mouse serum. (**A**) PCA score plot of the serum samples in control, *H. pylori*, *H. pylori*-low-dose, and *H. pylori*-high-dose groups. (**B**) Up- and downregulated differential metabolites between the control group and *H. pylori* group. (**C**) Stacked bar chart of the percentage of differential metabolites between the control group and the *H. pylori* group among all detected metabolites. (**D**) Pairwise comparisons of the top 30 differential serum metabolites with the largest absolute values of log_2_FC between the control group and the *H. pylori* group. The color gradient denotes Spearman’s correlation coefficients. The liver and stomach organ coefficients were related to each metabolite by Mantel tests. Line width corresponds to the Mantel’s r statistic for the corresponding distance correlations, and line color denotes the statistical significance.

The mouse serum metabolomics data were integrated with lipidomics data to obtain more comprehensive metabolome data for subsequent analysis. Firstly, to assess the differences in the serum metabolome among different groups of mice, PCA was employed for data dimensionality reduction. As shown in Figure 3A, the PCA score plot demonstrates significant distinctions among the four groups, indicating that *H. pylori* infection can cause substantial alterations in serum metabolites. Additionally, intervention with sulforaphane can partially alter the serum metabolic profile of *H. pylori*-infected mice. Differential metabolites between the control group and the *H. pylori* group were identified using independent samples *t*-tests, (FDR-adjusted *p*-values < 0.05). A total of 242 differential metabolites were identified, accounting for 25% of the total detected metabolites. Moreover, the majority of the differential metabolites were significantly downregulated (Figure S4). As shown in Figure 3B, the top five categories of differential metabolites with the highest number of downregulated metabolites were triglycerides (TGs), amino acids, phosphatidylcholine (PC), fatty acids, and acylcarnitines. The analysis of the percentage of differential metabolites within each class relative to the total number of detected metabolites in that class revealed that *H. pylori* infection may primarily influence the levels of acylcarnitines in serum (Figure 3C). The top 30 serum differential metabolites exhibiting the largest absolute values of log_2_ fold change (log_2_FC) between the control group and the *H. pylori* group were selected for correlation analysis with the liver and stomach organ coefficients using a Mantel test. As shown in Figure 3D, significant positive correlations between the differential metabolites and liver organ coefficients were observed. These findings suggest that the trend of change in liver coefficients is synergistic with the trend of change in levels of these key differential metabolites, primarily lipids, amino acids, and their derivatives, within the sample population. This alignment indicates a potential association between these metabolites and the physiological status of the liver.

**Figure 4:**
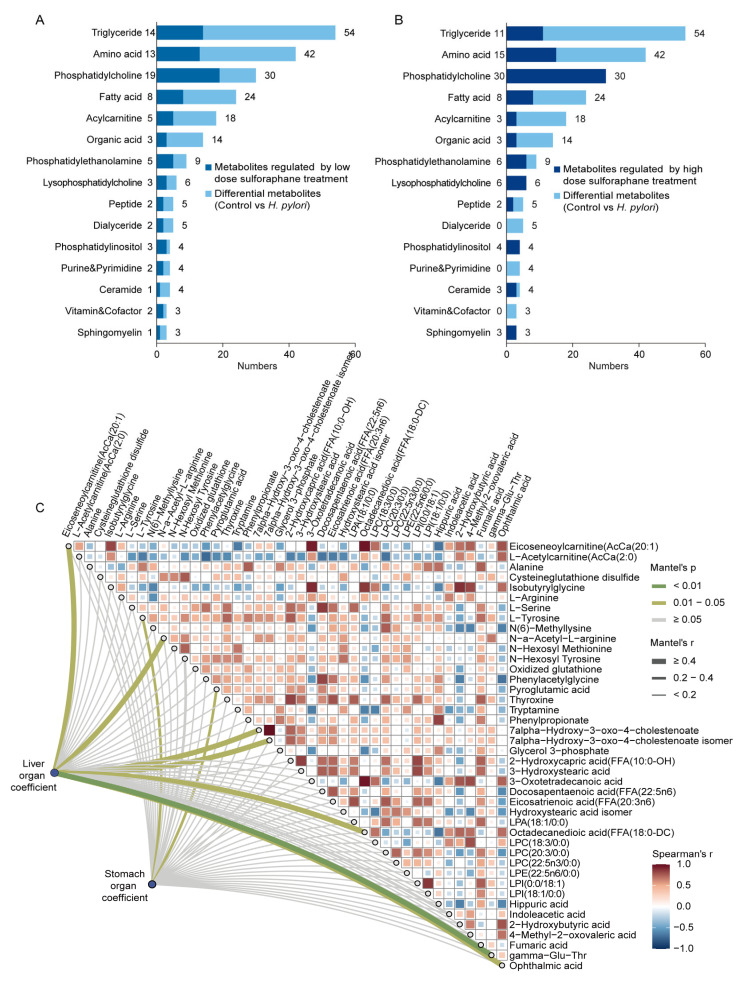
Metabolomics and lipidomics analysis of the effects of sulforaphane treatment on mouse serum. Stacked bar chart of the metabolites regulated by (**A**) low-dose and (**B**) high-dose sulforaphane treatment compared to the differential metabolites between the control group and *H. pylori* group. *H. pylori*-low-dose and *H. pylori*-high-dose represent 4 weeks of gavage with 5 mg/kg/d and 20 mg/kg/d of sulforaphane after *H. pylori* colonization in mice, respectively. (**C**) Pairwise comparisons of the differential serum metabolites between the control group and the *H. pylori* group which can be regulated by high-dose sulforaphane based on metabolomics data. The color gradient denotes Spearman’s correlation coefficients. The liver and stomach organ coefficients were related to each metabolite by Mantel tests. Line width corresponds to the Mantel’s r statistic for the corresponding distance correlations, and line color denotes the statistical significance.

For the differential serum metabolites between the control group and the *H. pylori* group, particular attention was given to the top 15 most abundant metabolite classes to identify the primary metabolites that sulforaphane treatment can regulate. Among these differential metabolites, the metabolites that did not show significant differences from the control in either the *H. pylori*-low-dose or *H. pylori*-high-dose group, as well as those that differed between the *H. pylori*-low-dose or *H. pylori*-high-dose group and the *H. pylori* group, were further screened out to identify the serum metabolites that can be influenced by sulforaphane intervention. As shown in Figure 4A, low-dose sulforaphane treatment can reverse 93 differential metabolites, accounting for 38% of the differential metabolites in mouse serum between the control group and the *H. pylori* group. The top three classes of reversed metabolites were TG, amino acids, and PC. In comparison to the *H. pylori*-low-dose group, treatment with a high dose of sulforaphane exhibited a more pronounced regulatory effect, reversing 43% of the differential metabolites between the control group and the *H. pylori* group. The effects on triglycerides and amino acids were comparable to those observed in the *H. pylori*-low-dose group. However, the high dose treatment demonstrated a greater capacity to regulate PC and lysophosphatidylcholine (LPC) species (Figure 4B).

We further investigated the relationships between sulforaphane-regulated serum molecules and organ (liver and stomach) coefficients using integrated metabolomic and lipidomic data. Under low-dose sulforaphane, metabolomic analysis (Figure S5) revealed strong inter-correlations among multiple short- and medium-chain acylcarnitines (AcCa(2:0), AcCa(6:0), AcCa(8:0)) and several free fatty acids (FFAs). These low-dose-regulated metabolites exhibited a stronger correlation with stomach organ coefficients than with liver organ coefficients. Lipidomic analysis (Figure S6) similarly showed strong inter-correlations among various phosphatidylcholines (PCs), phosphatidylethanolamines (PEs), and triacylglycerols (TGs). In contrast to the metabolites, these lipids regulated by low-dose sulforaphane correlated more strongly with liver organ coefficients.

High-dose sulforaphane treatment led to distinct correlational patterns. Seven serum metabolites, namely eicosenoylcarnitine (AcCa(20:1)), *N*-alpha-acetyl-L-arginine, octadecanedioic acid, bile acids, and peptides, were significantly and positively correlated with liver organ coefficients (Figure 4C). This suggests a synergistic relationship between changes in liver coefficients and the levels of these metabolites, implying their association with the liver’s pathophysiological state under this treatment. Additionally, L-tyrosine and pyroglutamic acid showed significant positive correlations with stomach organ coefficients, indicating a similar link between these two metabolites and the stomach’s pathophysiological state. Furthermore, lipidomic analysis revealed that five serum lipids regulated by high-dose sulforaphane (PC(40:4)-PC(20:0/20:4), TG(46:0)-TG(16:0/14:0/16:0), TG(48:2)-TG(16:0/14:0/18:2), TG(52:7)-TG(14:0/18:2/20:5), and TG(56:10)-TG(18:3/18:2/20:5)) were significantly and positively correlated with stomach organ coefficients (Figure S7). This points to an association between these lipids and the stomach’s pathophysiological condition as influenced by high-dose sulforaphane.

### 2.4. Mouse Liver Metabolomics and Lipidomics Analysis

**Figure 5:**
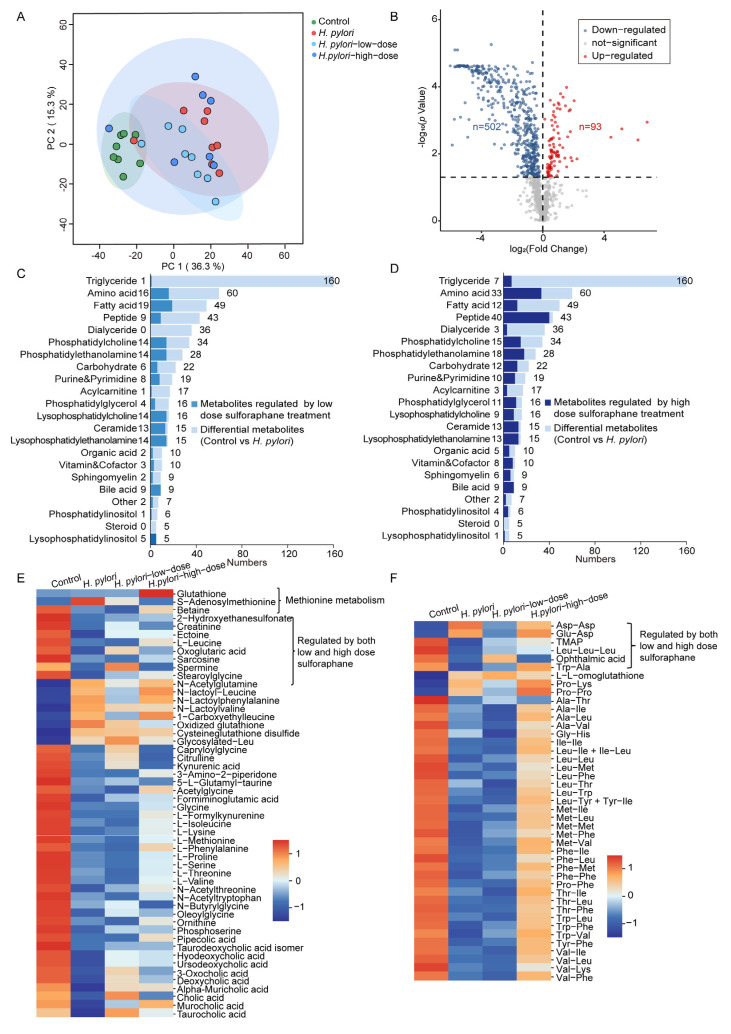
Mouse liver metabolomics and lipidomics analysis. (**A**) PCA score plot of the liver samples in control, *H. pylori*, *H. pylori*-low-dose, and *H. pylori*-high-dose groups. (**B**) Volcano plots of differential metabolites in the liver for the control and *H. pylori* groups. Stacked bar plot of the metabolites regulated by (**C**) low-dose and (**D**) high-dose sulforaphane treatment compared to the differential metabolites between the control group and *H. pylori* group. (**E**) Heatmap of amino acids and bile acids in mice livers. (**F**) Heatmap of peptides in mice livers. *H. pylori*-low-dose and *H. pylori*-high-dose represent 4 weeks of gavage with 5 mg/kg/d and 20 mg/kg/d of sulforaphane after *H. pylori* colonization in mice, respectively.

The mouse liver metabolomics data were further integrated with lipidomics data to elucidate changes in liver metabolites. The PCA score plot in Figure 5A shows that *H. pylori* infection can lead to significant changes in liver metabolites, which are also partially modulated by the sulforaphane intervention. Further analysis revealed 594 differential metabolites in mouse liver between the control and *H. pylori* groups, accounting for 48% of the total detected metabolites. As shown in the volcano plot (Figure 5B), the majority of differential metabolites were significantly downregulated in the liver of *H. pylori*-infected mice. The differential metabolites were categorized, revealing that the top five significantly altered metabolite categories were triglycerides, amino acids, fatty acids, peptides, and diglycerides (Figure S10). Further calculation of the percentage of differential metabolites in each category relative to the total detected metabolites in that category revealed that *H. pylori* infection primarily affected the metabolic homeostasis of diglycerides and TG in mouse liver (Figure S9).

For the differential metabolites in the liver of mice between the control group and the *H. pylori* group, metabolite classes with ≤1 species were removed, and the emphasis was placed on metabolite classes with a relatively higher number of altered metabolites. Among the differential liver metabolites identified between the control and *H. pylori* group, those that did not differ from the control in either the low- or high-dose groups, as well as those that differed between the *H. pylori*-low-dose or *H. pylori*-high-dose group and the *H. pylori* group, were further screened out to discover the metabolites that can be altered by sulforaphane intervention in the liver. A low dose of sulforaphane treatment can reverse 157 differential metabolites, accounting for 26% of the differential metabolites between the control group and *H. pylori* group. Among them, 50% of the PE, 87% of the LPC and ceramides, and 95% of the lysophosphatidylethanolamine (LPE) species, as well as all bile acids and lysophosphatidylinositol (LPI) species, were reversed (Figure 5C). However, the regulatory effects on other classes of metabolites were relatively weak. Compared to the *H. pylori*-low-dose group, high-dose sulforaphane intervention had better regulatory effects and can reverse 40% of the differential metabolites caused by *H. pylori* infection. It can regulate over 50% of amino acids and PE, carbohydrate, purine and pyrimidine, phosphatidylglycerol, LPC, ceramide, LPE, vitamin and cofactor, sphingomyelin (SM), and phosphatidylinositol (PI) species (Figure 5D). Notably, the bile acids can also be effectively adjusted. Furthermore, the high dose of sulforaphane treatment can significantly reverse more peptide species compared to the *H. pylori*-low-dose group. The heatmap illustrated the relative content distribution of differential metabolites, including amino acids, peptides, and bile acids, across the control, *H. pylori*, *H. pylori*-low-dose, and *H. pylori*-high-dose groups (Figure 5E). Among them, glutathione, S-adenosyl methionine, and betaine are involved in methionine metabolism. Additionally, nine amino acids and their derivatives (Figure 5E) and six peptides (Figure 5F) can be regulated by both low and high doses of sulforaphane treatment.

### 2.5. Metabolic Pathway Analysis and Proteomic Analysis

**Figure 6:**
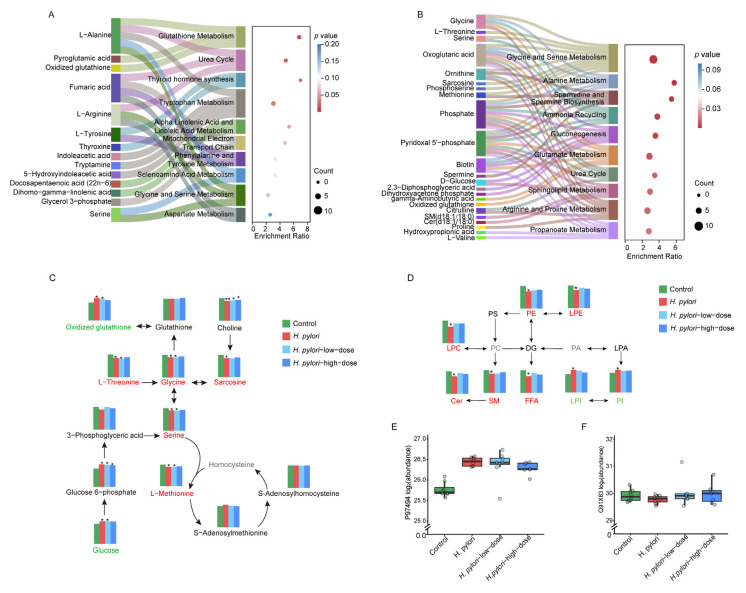
Metabolic pathway analysis. Pathway enrichment analysis results of metabolites regulated by sulforaphane treatment in the (**A**) serum and (**B**) liver. (**C**) Modulation of glutathione, methionine, and glycolysis-related metabolic pathways in mouse liver by sulforaphane treatment. (**D**) Regulation of lipid levels in the mouse liver by sulforaphane treatment. (**E**) P97494 log_2_FC abundance. (**F**) Q91X83 log_2_FC abundance. Red font indicates the metabolite decreases after *H. pylori* infection, followed by an increase after sulforaphane intervention; green indicates the metabolite increases after *H. pylori* infection, followed by a decrease after sulforaphane intervention; black indicates no significant changes; grey indicates that the substance was not detected. Abbreviations: LPC for lysophosphatidylcholine, PC for phosphatidylcholine, PS for phosphatidylserine, PE for phosphatidylethanolamine, LPE for lysophosphatidylethanolamine, SM for sphingomyelin, Cer for ceramide, PA for phosphatidic acid, LPA for lysophosphatidic acid, DG for diglyceride, FFA for free fatty acid, PI for phosphatidylinositol, LPI for lysophosphatidylinositol. *H. pylori*-low-dose and *H. pylori*-high-dose represent 4 weeks of gavage with 5 mg/kg/d and 20 mg/kg/d of sulforaphane after *H. pylori* colonization in mice, respectively. “*” indicates comparison with the control group, *p* ≤ 0.05; “**” indicates comparison with the control group, *p* ≤ 0.01.

The differential metabolites in the serum and liver that can be reversed by sulforaphane treatment were subjected to pathway enrichment analysis to identify the associated metabolic pathways. The results showed that the differential metabolites reversed by sulforaphane in serum were primarily related to pathways including glutathione metabolism, urea cycle, thyroid hormone synthesis, and tryptophan metabolism. Furthermore, changes in alanine levels were associated with multiple pathways (Figure 6A). In the liver, the differential metabolites regulated by sulforaphane treatment were predominantly related to pathways including glycine and serine metabolism, alanine metabolism, spermidine and spermine biosynthesis, ammonia recycling, and gluconeogenesis. Additionally, glycine, oxoglutaric acid, phosphate, pyridoxal 5′-phosphate, and biotin were linked to multiple pathways (Figure 6B). Sulforaphane treatment can upregulate glycine, serine, and L-methionine levels while downregulating both glucose and oxidized glutathione levels in *H. pylori*-infected mice (Figure 6C). In addition, sulforaphane also demonstrated a regulatory effect on lipid metabolism disorders caused by *H. pylori* infection in the mouse liver. As shown in Figure 6D, sulforaphane treatment can upregulate the levels of LPC, SM, ceramide (Cer), PE, LPE, and free fatty acids in the liver of *H. pylori*-infected mice, while the levels of PI and LPI were downregulated.

We further validated the metabolic function of glutathione and the biosynthetic function of glycine using proteomic analysis. As shown in Figure 6E,F, the expression of the key rate-limiting enzyme for glutathione synthesis (GGLC, P97494) was upregulated following *H*. *pylori* infection, while sulforaphane intervention significantly reduced its expression. Furthermore, *H*. *pylori* infection led to the downregulation of the expression of sarcosine dehydrogenase (SARDH, Q91X83), a key enzyme in the final step of glycine synthesis, which was reversed by sulforaphane intervention.

## 3. Discussion

Metabolomics and lipidomics provide extensive coverage of polar metabolites and non-polar lipids, enabling an accurate understanding of the host metabolic state. Combined with histopathological and oxidative stress marker analysis, these approaches systematically evaluate the pathogenic characteristics of *H. pylori* and the effects of sulforaphane intervention. Sulforaphane treatment significantly alleviates gastric damage and increases the levels of antioxidants. Moreover, sulforaphane demonstrates a significant regulatory effect on *H. pylori*-induced metabolic disorders, particularly in amino acids, bile acids, glycerophospholipids, ceramides, and peptides.

### 3.1. Therapeutic Effects of Sulforaphane on Stomach and Liver

*H. pylori* infection is found to adhere to and invade the stomach tissue of mice (Figure S1). H&E staining of stomach sections revealed that *H. pylori* infection can induce degeneration of the epithelial cells in the gastric mucosa (Figure 1F). Sulforaphane treatment alleviated the vacuolar degeneration of gastric mucosa epithelial cells in a dose-dependent manner (Figure 1G,H). *H. pylori* infection also can induce oxidative stress in the stomach [13]. GSH and SOD are involved in the detoxification of reactive oxygen species. *H. pylori* infection decreased both T-GSH content and SOD activity in the stomach of infected mice. Sulforaphane intervention significantly upregulated T-GSH content and SOD activity (Figure 1I,J). In this study, IL-18 mRNA expression was upregulated after sulforaphane treatment (Figure 1K). Our speculation about its protective role is mainly based on two points: first, previous studies have elucidated that, in response to *H. pylori*, gastric epithelial cells produce IL-18 through a specific NOD1-mediated pathway that is critical for maintaining epithelial homeostasis and protecting against infection-induced pathology [14]. This is consistent with the trend of sulforaphane alleviating gastric mucosal vacuolar degeneration in our study (Figure 1G,H). Second, if the upregulation of IL-18 were proinflammatory, it would typically be accompanied by more severe tissue damage (e.g., aggravated inflammatory cell infiltration). However, we observed that sulforaphane treatment alleviated gastric mucosal pathological damage, with significant improvements in oxidative stress markers (T-GSH, SOD) (Figure 1I,J). This phenomenon of “elevated IL-18 accompanied by reduced damage” is more consistent with its protective role—i.e., moderately activating immune responses to clear pathogens rather than causing tissue destruction via excessive inflammation. While we concede that additional cytokine data would offer a broader view, the existing pathological evidence, supported by relevant literature, points toward the sulforaphane-induced IL-18 expression being a beneficial host mechanism.

Furthermore, this study found that *H. pylori* infection significantly increased both liver weight and liver organ coefficients of mice. Previous research has reported that the *H. pylori* strain SS1 can cause liver inflammation in C57BL/6 mice [15], which is consistent with our findings. Interestingly, both low and high doses of sulforaphane treatment can reduce the liver weight and liver organ coefficient, suggesting that the liver may serve as a key target organ for sulforaphane.

### 3.2. Sulforaphane Restores Amino Acid and Glutathione Metabolism Disrupted by H. pylori

The metabolomic analysis revealed that *H. pylori* infection can cause significant alterations in serum amino acid levels of mice (Figure 3B) and substantial downregulation of amino acids and peptides in the liver (Figure S8). Among these, glycine was a key metabolite of interest. Its levels were markedly decreased in the livers of *H. pylori*-infected mice, whereas treatment with a high dose of sulforaphane effectively restored them (Figure 5E, Figure 6C and Figure S10A). Glycine is essential for host defense, as its administration has been shown to reduce bacterial load in infected mice, suggesting it plays a significant role in suppressing bacterial infections [16,17].

Given glycine’s critical role as a precursor for GSH biosynthesis [18], we investigated the impact of its depletion on the host’s antioxidant system. We observed elevated levels of oxidized glutathione (GSSG) in the livers of infected mice, an indicator of severe oxidative stress. Sulforaphane treatment dose-dependently downregulated GSSG (Figure S10D) and significantly upregulated T-GSH levels in the stomach (Figure 1I), highlighting its capacity to restore redox balance. Pathway enrichment analysis further confirmed that metabolites regulated by sulforaphane in serum were enriched in the glutathione metabolic pathway (Figure 6A).

To understand the molecular machinery behind these observations, our proteomic data provided key mechanistic insights. We found a paradoxical upregulation of GCLC, the rate-limiting enzyme for GSH synthesis, in the infected group (logFC = 0.67). We interpret this as a compensatory host defense response to counteract severe oxidative stress. However, despite this adaptation, the GSH pool remained depleted, indicating overwhelming demand or, crucially, substrate limitation due to glycine depletion. This context highlights the efficacy of sulforaphane. Low-dose treatment downregulated GCLC expression relative to the infected group (logFC = −0.07), while high-dose sulforaphane further restored GCLC expression toward baseline (logFC = −0.15 relative to infected group), suggesting a reestablishment of redox homeostasis that obviated the need for a sustained compensatory response.

This identified substrate availability as a critical bottleneck, leading us to investigate the glycine synthesis pathway. Glycine can be synthesized from choline [18], and our data showed that, while infection decreased liver choline, sulforaphane treatment increased the levels of choline, betaine, and sarcosine (Figure 6C and Figure S10B,C). Our proteomic analysis offers strong functional validation for this pathway’s role. We observed that, while *H. pylori* infection slightly suppressed SARDH—the enzyme catalyzing the terminal step of this pathway—high-dose sulforaphane treatment reversed this, upregulating its expression (logFC = 0.20). This provides direct evidence that sulforaphane enhances the enzymatic machinery for glycine precursor synthesis.

Collectively, these combined proteomic and metabolomic findings provide robust mechanistic validation for our observations, revealing a sophisticated dual action of sulforaphane: it not only enhances the primary defense via GCLC but also secures the substrate supply for this defense via SARDH.

Furthermore, sulforaphane’s regulatory effects extended to other related amino acids. The hepatic levels of serine and threonine, which can also contribute to glycine metabolism, were decreased by infection and restored by high-dose sulforaphane (Figure 6C and Figure S10C). Similarly, sulforaphane reversed the infection-induced decrease in dipeptides (Figure 5D), which may contribute to antioxidant processes, as certain dipeptides have been shown to elevate glutathione levels and activity [19,20]. These additional findings underscore the comprehensive role of sulforaphane in restoring metabolic homeostasis to combat *H. pylori*-induced pathology.

### 3.3. Bile-Acid-Related Metabolism

Similar to the alterations in amino acid metabolism, liver bile acid metabolism also exhibited significant downregulation following *H. pylori* infection. Primary bile acids synthesized in the liver are converted into secondary bile acids by gut microbiota and can be conjugated with glycine or taurine to form conjugated bile acids [21]. This study found that hepatic levels of cholic acid and chenodeoxycholic acid were significantly decreased post-infection. This reduction may inhibit the activation of the farnesoid X receptor (FXR), leading to dysregulation of the CYP7A1-mediated negative feedback loop for bile acid synthesis and consequently contributing to bile acid pool disruption [22]. Concurrently, the decrease in hyodeoxycholic acid may further disrupt gut microbial balance, impairing its ability to suppress pathogenic bacterial proliferation and regulate lipolytic metabolism [23]. Additionally, reduced levels of ursodeoxycholic acid may affect the expression of intestinal tight junction proteins, exacerbating gut barrier damage [24]. Notably, gut microbiota homeostasis is closely linked to tryptophan metabolism, and the two engage in bidirectional regulation through the “bile acid–aryl hydrocarbon receptor (AhR)–microbiota” network [25]. Our results demonstrate that both low-dose and high-dose sulforaphane interventions effectively normalized the levels of all differentially expressed bile acid metabolites and reversed the abnormal elevation of kynurenic acid. Based on these findings, we hypothesize that sulforaphane may restore metabolic homeostasis through multi-target actions: on one hand, by promoting bile acid conjugation to selectively enrich specific probiotics; on the other hand, by activating the AhR-Nrf2 signaling pathway to enhance intestinal barrier function, thereby disrupting the “leaky gut–lipopolysaccharide translocation–liver inflammation” vicious cycle. Moreover, existing studies have found that sulforaphane can regulate intestinal flora and enhance the ability of beneficial bacteria to alleviate liver metabolic disorders [26].

### 3.4. Lipid-Related Metabolism

Glycerophospholipid is one of the main components of the cell membrane involved in cell signal transduction. Among them, PE is one of the most abundant phospholipids in mammalian cells, showing significant changes in the development of various diseases [12]. Phospholipid metabolism is associated with autoimmune regulation and inflammatory responses [27]. Balonov et al. conducted a systematic review and meta-analysis of metabolite markers for the identification of upper gastrointestinal cancer, and they found that the levels of PC in patients with upper gastrointestinal cancer were significantly higher than those in normal individuals [28]. Study also found LPC is negatively correlated with the occurrence and development of gastric diseases [29]. In our study, it was found that, after *H. pylori* infection, 11 and 20 differential lipids of the PC class showed an increasing trend in the serum and liver, while 1 and 13 differential lipids of the LPC class were significantly downregulated (Figure 3B and Figure S8). High-dose sulforaphane treatment showed a regulatory effect on all serum differential metabolites in the PC and LPC classes (Figure 4B). Sulforaphane can also reverse the increased levels of PC in the liver after *H. pylori* infection (Figure 6D). Based on these results, it can be inferred that sulforaphane has strong regulatory effects on the phospholipid metabolic pathways, which are closely related to gastric diseases caused by *H. pylori* infection.

Ceramides can regulate the immune function of macrophages and play important roles in participating in various signal transduction pathways related to cell survival and aging [30]. Sphingolipid metabolism, in which ceramide and SM are involved, is closely related to inflammatory reactions in cells [12]. In this study, sulforaphane treatment can reverse the changes in ceramide and sphingomyelin levels in the liver of *H. pylori*-infected mice. This suggests that sulforaphane may have a role in improving metabolic disorders of SM and thus preventing inflammation.

Triglycerides were significantly downregulated in both mouse serum and liver after *H. pylori* infection. However, the available evidence on the relationship between *H. pylori* infection and triglyceride is limited and conflicting. In a study by Liu et al., it was found that *H. pylori* infection is not significantly associated with triglyceride levels [31]. Whereas in the study by Haeri et al., it was found that *H. pylori* seropositive female patients had lower serum triglyceride levels [32]. On the contrary, another study reported a positive correlation between triglyceride levels in female serum and *H. pylori* seropositivity [33]. Since this study used male mice, it is speculated that the differing changes in triglyceride levels may be related to gender factors.

### 3.5. Energy Metabolism

Glucose metabolism pathways are pivotal in the energy metabolism of mice. The pathway enrichment analysis of liver metabolites demonstrated that the metabolites regulated by sulforaphane treatment are involved in glucose-metabolism-related pathways, including alanine metabolism and the gluconeogenesis pathway (Figure 6B). Pathway enrichment analysis of serum metabolites also indicated multiple pathways associated with alanine (Figure 6A). Alanine serves as a precursor for hepatic gluconeogenesis, while glucose can be converted into pyruvate through glycolysis [34]. Glycolysis and gluconeogenesis are vital pathways in glucose metabolism [35]. Our results revealed that sulforaphane can significantly reduce the levels of glucose and glucose 6-phosphate in the livers of *H. pylori*-infected mice (Figure 6C), suggesting a close relationship between sulforaphane and glucose-related energy metabolism.

## 4. Materials and Methods

### 4.1. Reagents and Instruments

Sulforaphane standard (95% purity, CAS: 4478-93-7) was purchased from Shanghai Aladdin Biochemical Technology Co., Ltd. (Shanghai, China). Dimethyl sulfoxide (No. D806645) was purchased from Shanghai McLean Biochemical Technology Co., Ltd. (Shanghai, China). *Helicobacter pylori* 26,695 was cultured in Columbia medium under microaerobic conditions (6% O_2_, 7.2% CO_2_, 7.1% H_2_, 79.7% N_2_) for 24 h. Methanol, acetonitrile, and formic acid were purchased from Fisher Scientific (Fair Lawn, NJ, USA). Ultra-pure water (18.2 MΩ·cm) was prepared using a Milli-Q water purification system (Merck KGaA, Darmstadt, Germany).

A centrifuge was purchased from Hunan Hengnuo Instrument and Equipment Co., Ltd. (Changsha, Hunan, China). A grinder was purchased from Wuhan Sevier Biotechnology Co., Ltd. (Wuhan, Hubei, China). The UHPLC-HRMS system used in this study was an UltiMate 3000 ultra-high-performance liquid chromatography instrument coupled to a Q Exactive high-resolution mass spectrometer (Thermo Scientific, San Jose, CA, USA).

### 4.2. Animal Experiment

Six-week-old male C57BL/6J mice were purchased from Shanghai SLAC Laboratory Animal Co., Ltd. (Shanghai, China). All mice were housed and received interventions in the Experimental Animal Center of Fujian Medical University during the experiment. The mice were housed at a room temperature of 23 ± 2 °C and 45 ± 10% relative humidity and under a 12/12 h light/dark cycle with ad libitum food and water intake. All animal experimental procedures were approved by the Animal Ethics Committee of Fujian Medical University (IACUC FJMU 2022-0841).

After one week of acclimatization feeding, the mice were randomly divided into 4 groups: control group (*n* = 8), *H. pylori* group (*n* = 9), *H. pylori*-low-dose group (*n* = 8), and *H. pylori*-high-dose group (*n* = 8). One mouse from the *H. pylori* group was used for *H. pylori* colonization identification. The sample size of 8 mice per group was determined in accordance with the 3Rs principles and the resource equation method. This size was deemed sufficient to ensure adequate statistical power, account for potential sample loss and biological variability, while also meeting the minimum requirement of at least four biological replicates for metabolomics analysis. To induce normal gastric epithelial apoptosis and facilitate the colonization of *H. pylori*, we used *H. pylori* and *N*-methyl-*N*’-nitro-*N*-nitrosoguanidine (MNNG) to establish an *H. pylori*-infected mouse model. Except for the control group, all other groups were given free access to drinking water containing MNNG (100 mg/L) and orally gavaged 400 μL of 10^9^ CFU/mL of *H. pylori* once a week for 4 weeks, totaling 4 administrations. After 4 weeks, one mouse was randomly selected from the *H. pylori* group, and its gastric mucosal tissues were harvested by autopsy to identify the presence of *H. pylori*. The sulforaphane intervention was then carried out. The specific treatments for each group were as follows: the control group was orally gavaged with 0.9% saline (0.3 mL/mouse/day); the *H. pylori* group was orally gavaged with 0.9% saline (0.3 mL/mouse/day); the *H. pylori*-low-dose group was orally gavaged with 5 mg/kg of sulforaphane per day; and the *H. pylori*-high-dose group was orally gavaged with 20 mg/kg of sulforaphane per day. The 20 mg/kg dose was chosen as it aligns with a dose shown to be highly effective in attenuating gastritis in murine models of *H. pylori* infection (approx. 21 mg/kg) and corresponds to levels used in key pharmacokinetic studies characterizing sulforaphane’s distribution in the liver [36,37]. The 5 mg/kg dose was selected as a mechanistic probe, as it is an established concentration for specifically activating the NRF2-dependent antioxidant pathway while minimizing potential off-target effects [38]. Both doses are well within the established therapeutic window and are an order of magnitude below the reported median toxic dose (TD50 ≈ 192 mg/kg) in mice, ensuring that the observed metabolic effects are therapeutic and not a result of toxicity [39].

Throughout the experiment, the body weights, food intake, and water intake of the mice were recorded every 3 days, and the organ weights of the mice in each group were recorded at the time of sampling. After 4 weeks of the intervention, the mice were euthanized by decapitation, and the serum, liver, stomach, and other organs were collected. The weights of the organs were recorded and the samples were stored in a refrigerator at −80 °C for subsequent analysis. There were no exclusions of animals. The researchers were informed of the group allocations throughout the experiment and data analysis.

### 4.3. Assessment of H. pylori Colonization

After four rounds of gavage in the *H. pylori* group, one mouse was randomly selected, and its gastric mucosa tissue was collected post-sacrifice. The tissue was placed in a sterile mortar tube containing 200 μL of Brucella broth and ground for 3 min. The homogenate was then spread onto a blood agar plate and incubated under microaerobic conditions for 72 h. Suspected colonies were isolated and subcultured for an additional 72 h. Detection of these colonies was performed using a gastric *H. pylori* detection kit (Begen Biological Technology Co., Ltd., Sanming, Fujian, China).

### 4.4. Histological Evaluation

The stomachs of the mice were carefully excised and incised along the greater curvature. The samples were then immersed in a 4% paraformaldehyde solution for 24 h to ensure proper fixation. Following fixation, the tissues underwent a series of procedures, including dehydration, paraffin embedding, sectioning, and hematoxylin and eosin (H&E) staining. The sections were subsequently sealed and examined microscopically to evaluate the histopathological changes in the stomachs.

### 4.5. Determination of Oxidative Stress Indexes

To determine oxidative stress indexes, 10 mg of gastric tissue was weighed and homogenized in 190 μL of 0.9% NaCl aqueous solution using a homogenizer. The homogenized samples were centrifuged at 3500 rpm for 10 min at 4 °C to obtain the supernatant. Protein content, total glutathione (T-GSH) concentration, and superoxide dismutase (SOD) activity were measured using kits obtained from Nanjing Jianjian Bioengineering Institute Co. (Nanjing, China), following the manufacturer’s protocols.

### 4.6. RNA Extraction and Real-Time Quantitative RT-PCR

Total RNA was extracted from mouse gastric tissues using RNAiso Plus reagent (Takara Bio Inc., Kusatsu, Shiga, Japan). Reverse transcription was performed using the PrimeScriptRT reagent kit (Takara Bio Inc., Kusatsu, Shiga, Japan). Quantitative PCR analysis of the resulting cDNA was carried out using a SuperReal PreMix Plus (SYBR Green) kit (Tiangen Biotech Co., Ltd., Beijing, China) on the QuantStudio 5 Real-Time PCR System (Thermo Fisher Scientific Inc., Waltham, MA, USA). Primer sequences are provided in Table S1. Relative mRNA expression levels were calculated using the 2^−ΔΔCt^ method, with glyceraldehyde-3-phosphate dehydrogenase (GAPDH) mRNA serving as the endogenous reference control.

### 4.7. Sample Pre-Treatment

The liver tissue samples were thawed at room temperature, dissected with surgical scissors, and precisely weighed to obtain samples of 10.0 ± 1.0 mg. They were then added to 300 μL of 80% methanol–water solution and homogenized. After vortex mixing, 200 μL of tissue homogenate was taken and added with 480 μL of methyl tert-butyl ether and 120 μL of water, followed by vortex mixing for another 10 min. After centrifugation at 4 °C for 10 min, the upper layer of the extract was hydrophobic non-polar lipid components, while the lower layer extract was hydrophilic polar metabolic components. The serum samples were also thawed at room temperature, 200 μL of serum samples were taken and added with 480 μL of methyl tert-butyl ether and 120 μL of water, followed by vortex mixing for 10 min. The samples were centrifuged at 4 °C for 10 min. To ensure the reliability and reproducibility of sample processing and analysis, randomly selected samples were taken and mixed in equal amounts to serve as quality control (QC) samples. The QC samples were uniformly inserted at the beginning, middle, and end of the real samples’ data acquisition sequence to assess the instrument signal fluctuations and reproducibility of the experiment.

### 4.8. Non-Targeted Metabolomics and Lipidomics Analysis

The samples were analyzed by Dalian Hanku Medical Laboratory Co., Ltd. (Dalian, China). The analysis process followed the metabolomics and lipidomics analysis methods reported in the literature [40]. Metabolomics analysis was performed on the aforementioned UHPLC-HRMS system. Lipidomics analyses were performed on the same instrument operated in positive/negative polarity switching mode and lipid chromatographic separation was performed on an Accucore C30 core–shell column (Thermo Scientific, Bellefonte, PA, USA). Full-scan MS data at 70,000 FWHM resolution and the top 10 MS/MS data associated with the full-scan data were obtained using Xcalibur software (version 4.1, Thermo Scientific, San Jose, CA, USA). The metabolomics data were further processed with Compound Discoverer software (version 2.4, Thermo Scientific, San Jose, CA, USA) for comprehensive metabolite component extraction. The non-targeted lipidomics data (hydrophobic portion) was analyzed using Lipid Search software (version 4.4.20, Thermo Scientific, San Jose, CA, USA) for peak selection and lipid identification.

### 4.9. Proteomic Analysis

For proteomic profiling, proteins from mouse liver lysates were subjected to in-solution tryptic digestion. The resulting peptides were separated over a 60 min gradient using a Vanquish Neo UHPLC system (Thermo Fisher Scientific, Dreieich, Germany) and analyzed on an Orbitrap Exploris 480 mass spectrometer (Thermo Fisher Scientific, Dreieich, Germany). Data were acquired in data-independent acquisition (DIA) mode, and the resulting raw files were processed using DIA-NN (version 2.0).

### 4.10. Statistical Analysis

The line graph depicting body weight changes was plotted using GraphPad Prism software (version 9.5, GraphPad Software, San Jose, CA, USA). Data were analyzed using one-way analysis of variance (ANOVA) in SPSS (version 27, IBM Corp., Armonk, NY, USA), while the Kruskal–Wallis test was employed for non-normally distributed data. Half-violin plots of food intake, water intake, and organ coefficients were plotted using the R 4.4.2 packages “gghalves” and “ggplot2”. Box plots of oxidative stress indexes and inflammatory factors were plotted using the R package “ggplot2”. Principal component analysis (PCA) and the pathway enrichment analysis of metabolites altered by sulforaphane intervention were performed using MetaboAnalyst 6.0 [41] (https://www.metaboanalyst.ca/, accessed on 17 May 2024). Statistical analysis was performed using the *t*-test in SPSS software, and *p* < 0.05 was considered statistically significant after FDR correction. The volcano plot was plotted by using Hiplot [42] (https://hiplot.com.cn/, accessed on 5 February 2025). The heatmaps for visualization of key altered metabolites were plotted using OmicStudio [43] (https://www.omicstudio.cn/, accessed on 5 February 2025). The correlation between differential metabolites and liver and stomach organ coefficients were analyzed by the Mantel test using the R 4.4.2 package “linkET”.

## 5. Conclusions

In this study, UHPLC-HRMS-based high-coverage metabolomics and lipidomics analysis was conducted to investigate the regulatory effects of sulforaphane treatment on metabolic dysfunction caused by *H. pylori* infection in mice serum and liver. The results showed that *H. pylori* infection significantly altered host amino acid and lipid levels, specifically manifested as abnormal serum glycerophospholipids and metabolic imbalances of amino acids, bile acids, glycerophospholipids, ceramides, and peptides in the liver. Sulforaphane treatment reversed these metabolic abnormalities, with high-dose sulforaphane exhibiting more prominent regulatory effects. Notably, high-dose sulforaphane effectively restored hepatic metabolic disorders of amino acids, bile acids, and lipids, and ameliorated abnormal serum glycerophospholipid profiles. Mechanistically, the regulation of key pathways such as glycine metabolism and glutathione metabolism constitutes an important basis for sulforaphane’s anti-*H. pylori* infection effects, which are closely associated with the host’s antioxidant defense and anti-infective capacity. This study provides a comprehensive metabolic basis for understanding the role of sulforaphane as a dietary intervention in preventing and managing *H. pylori*-associated gastric diseases and lays a foundation for subsequent clinical translational research.

## Data Availability

The original contributions presented in this study are included in the article/Supplementary Materials. Further inquiries can be directed to the corresponding author(s).

## Supplementary Materials

The following supporting information can be downloaded at: https://www.mdpi.com/article/10.3390/ijms26167791/s1.
