# Supplementary material for: Therapeutic Effects of Sulforaphane on Helicobacter pylori-Infected Mice: Insights from High-Coverage Metabolomics and Lipidomics Analyses of Serum and Liver
Source: Int J Mol Sci. 2025 Aug 12;26(16):7791. doi: 10.3390/ijms26167791 (PMC12386524; doi:10.3390/ijms26167791)
Supplement: Supplementary file 1 [file ijms-26-07791-s001.zip › ijms-3742140-supplementary.pdf]

Supplementary Materials

Therapeutic Effects of Sulforaphane on *Helicobacter pylori*-Infected Mice: Insights from High-Coverage Metabolomics and Lipidomics Analyses of Serum and Liver

Shuling He [1,†], Lvyun Sun [1,†], Jiali Chen [1,†], Yixin Li [1], Ying Pan [1], Amei Su [1], Qiuyao Mao [1], Jiaqian Hu [1], Disheng Feng [2,*] and Yang Ouyang [1,*]

[1] Department of Health Inspection and Quarantine, School of Public Health, Fujian Medical University, Fuzhou 350122, China
[2] Fujian Key Laboratory of Chinese Materia Medica, Institute of Structural Pharmacology & TCM Chemical Biology, College of Pharmacy, Fujian University of Traditional Chinese Medicine, Fuzhou 350122, China
* Correspondence: fengds@fjtcm.edu.cn (D.F.); ouyangyang@fjmu.edu.cn (Y.O.)
† These authors contributed equally to this work.

Table of Contents

Table S1. Primer sequences used for real-time quantitative PCR.

Figure S1. Photographs of *H. pylori* detection results.

Figure S2. The half violin plots of organ coefficient column charts of mice.

Figure S3. Average water intake of mice in each group.

Figure S4. Differential metabolites analysis of mouse serum between the control and *H. pylori* groups.

Figure S5. Pairwise comparisons of the differential serum metabolites between the control group and the *H. pylori* group which can be regulated by low dose sulforaphane based on metabolomics data.

Figure S6. Pairwise comparisons of the differential serum lipids between the control group and the *H. pylori* group which can be regulated by low dose sulforaphane based on lipidomics data.

Figure S7. Pairwise comparisons of the differential serum lipids between the control group and the *H. pylori* group which can be regulated by high dose sulforaphane based on lipidomics data.

Figure S8. Up- and down-regulated differential metabolites between liver samples from the control group and *H. pylori* group.

Figure S9. Stacked bar chart of the percentage of differential metabolites in the total detected substances between liver samples from the control group and the *H. pylori* group.

Figure S10. Box plot of the relative concentrations in the liver.

Table S1. Primer sequences used for real-time quantitative PCR.

Gene	Primers	Sequences (5'-3')
**GAPDH**	Forward	AGGTCGGTGTGAACGGATTTG
	Reverse	TGTAGACCATGTAGTTGAGGTCA
**IL-18**	Forward	GACTCTTGCGTCAACTTCAAGG
	Reverse	CAGGCTGTCTTTTGTCAACGA

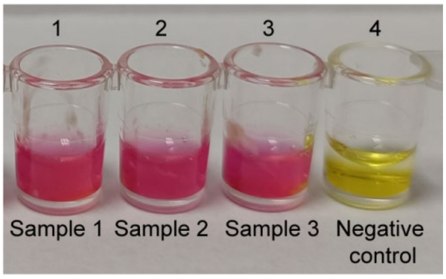

Figure S1. Photographs of *H. pylori* detection results of mouse gastric mucosa tissue. Well 1, 2, and 3 are sample assay wells, and 4 is the negative control.

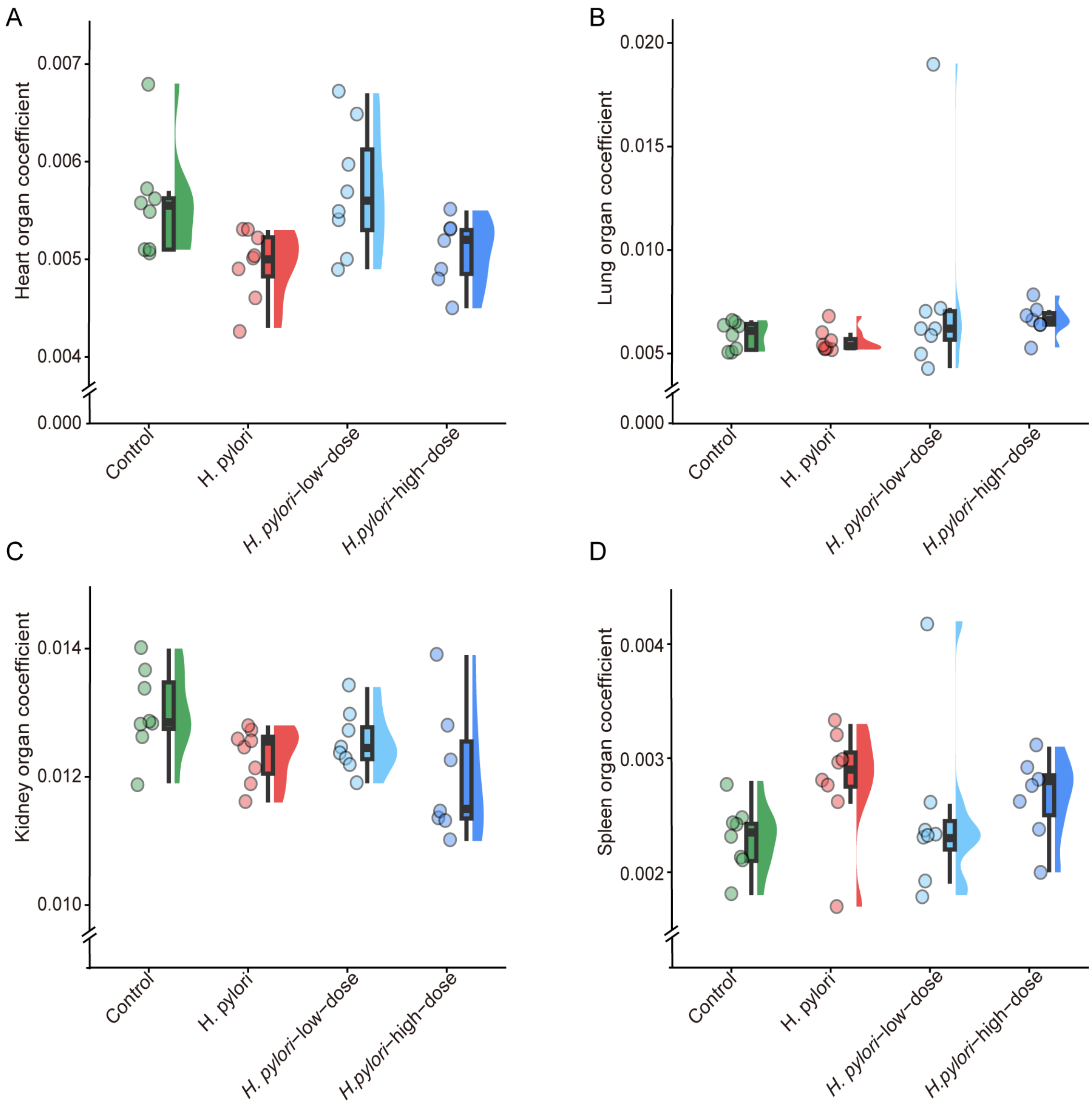

Figure S2. The half violin plots of organ coefficient column charts of mice. (A) heart (B) lung (C) kidney and (D) spleen in each grsoup. *H. pylori*-low-dose and *H. pylori*-high-dose represent 5 mg/kg/d and 20 mg/kg/d of sulforaphane gavage per day, respectively.

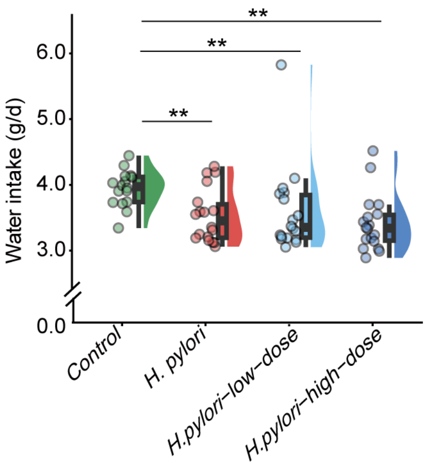

Figure S3. Average water intake of mice in each group. “**” means $p \leq 0.01$.

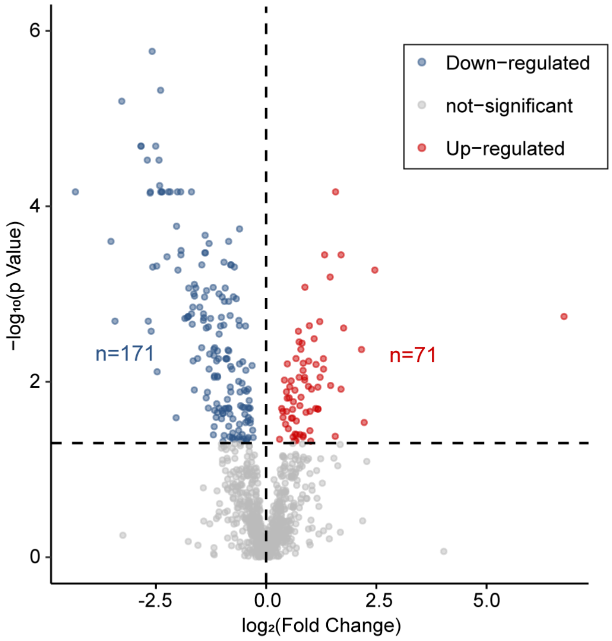

Figure S4. Differential metabolites analysis of mouse serum between the control and *H. pylori* groups. Metabolites with $\log_2$fold-change > 0 and p adj ≤ 0.05 are represented in red, and metabolites with $\log_2$fold-change < 0 and p adj ≤ 0.05 are represented in blue (based on t-test).

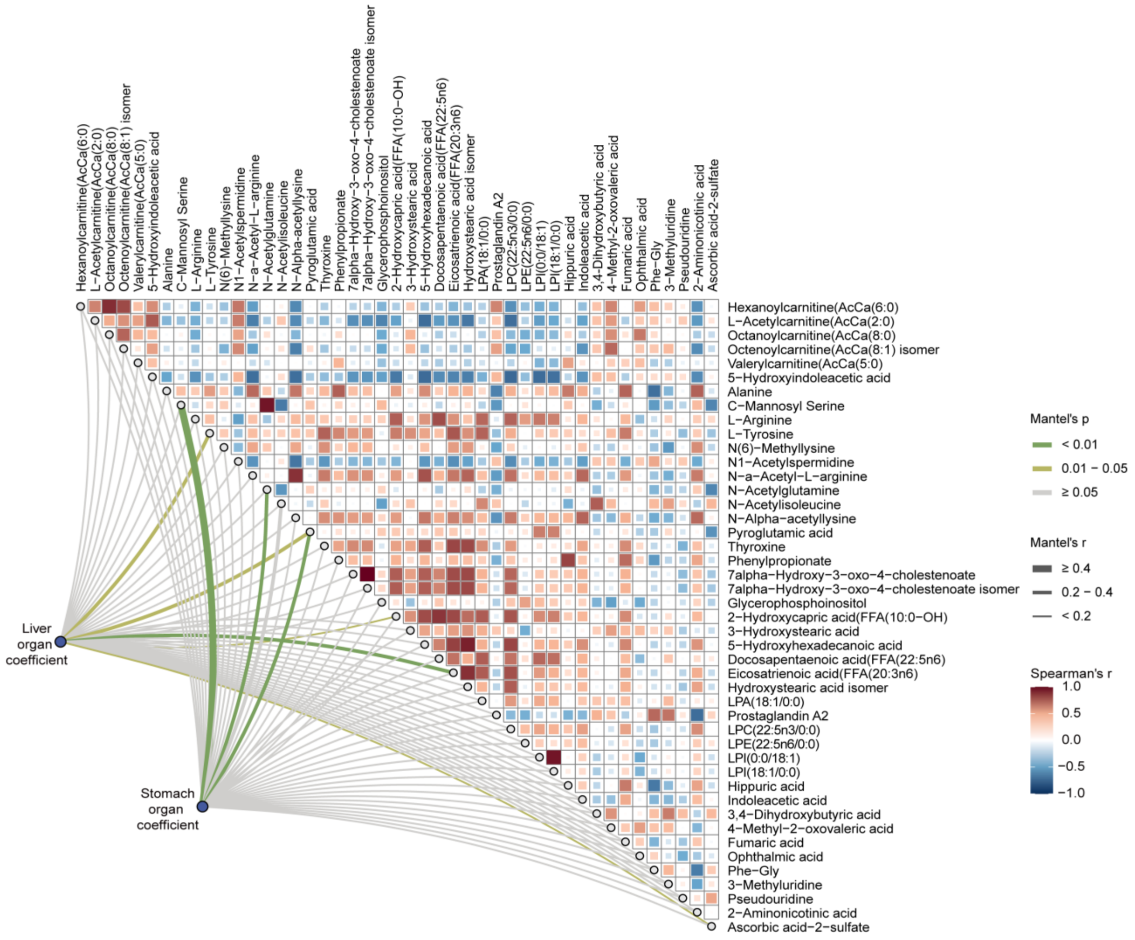

Figure S5. Pairwise comparisons of the differential serum metabolites between the control group and the *H. pylori* group which can be regulated by low dose sulforaphane based on metabolomics data, with a color gradient denoting Spearman's correlation coefficients. The liver and stomach organ coefficients distance matrix (Bray-Curtis dissimilarity) was related to each metabolite by Mantel tests. Edge width corresponds to the Mantel's r statistic for the corresponding distance correlations, and edge color denotes the statistical significance.

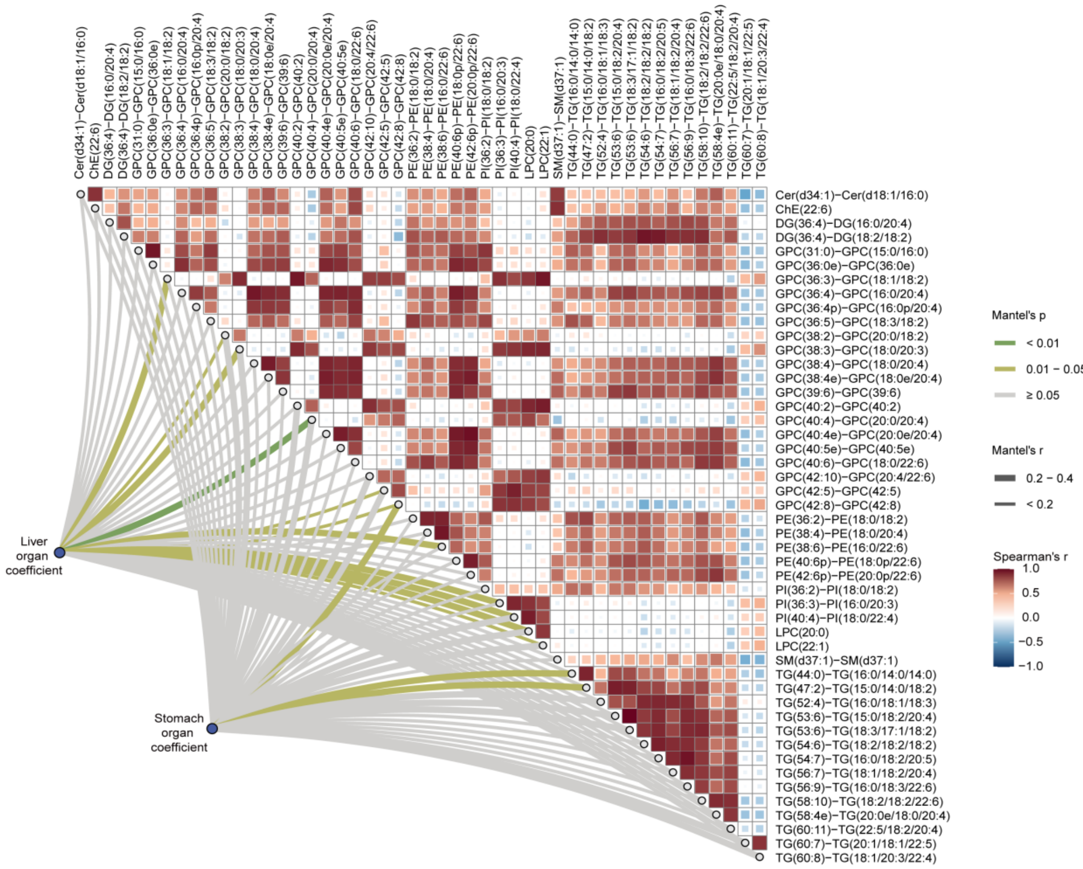

Figure S6. Pairwise comparisons of the differential serum lipids between the control group and the *H. pylori* group which can be regulated by low dose sulforaphane based on lipidomics data, with a color gradient denoting Spearman's correlation coefficients. The liver and stomach organ coefficients distance matrix (Bray-Curtis dissimilarity) was related to each metabolite by Mantel tests. Edge width corresponds to the Mantel's r statistic for the corresponding distance correlations, and edge color denotes the statistical significance.

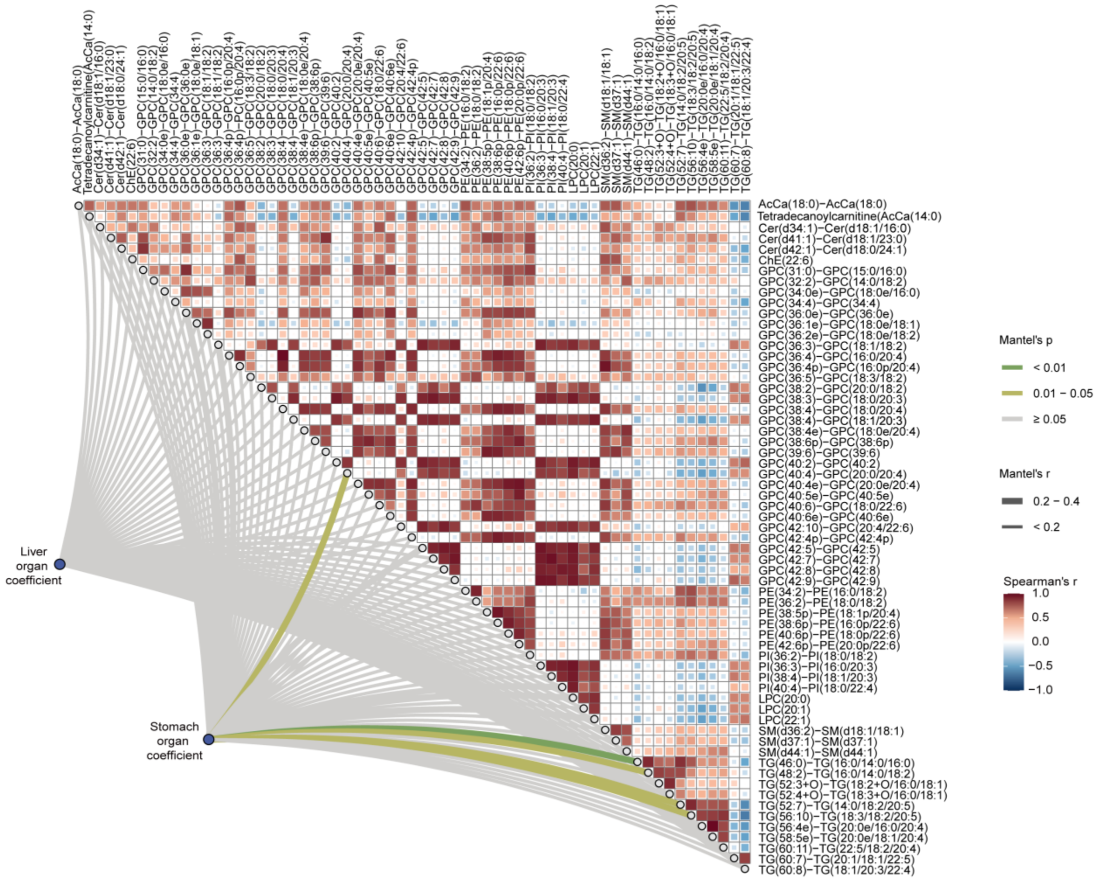

Figure S7. Pairwise comparisons of the differential serum lipids between the control group and the *H. pylori* group which can be regulated by high dose sulforaphane based on lipidomics data, with a color gradient denoting Spearman's correlation coefficients. The liver and stomach organ coefficients distance matrix (Bray-Curtis dissimilarity) was related to each metabolite by Mantel tests. Edge width corresponds to the Mantel's r statistic for the corresponding distance correlations, and edge color denotes the statistical significance.

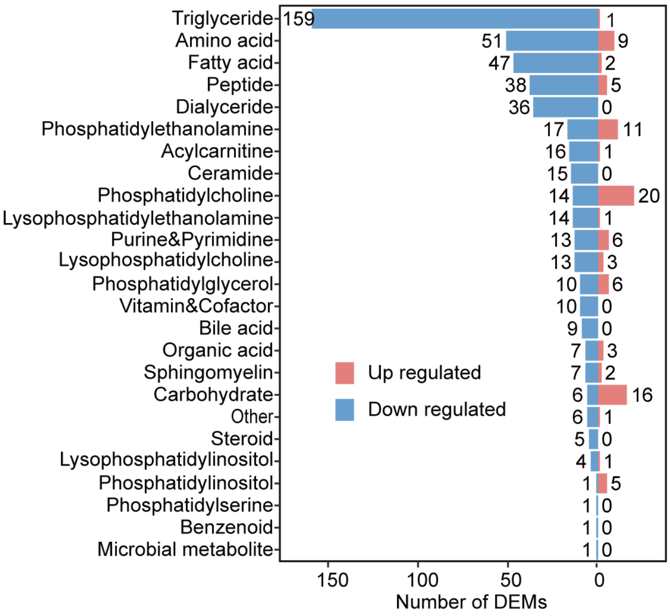

Figure S8. Up- and down-regulated differential metabolites between liver samples from the control group and *H. pylori* group.

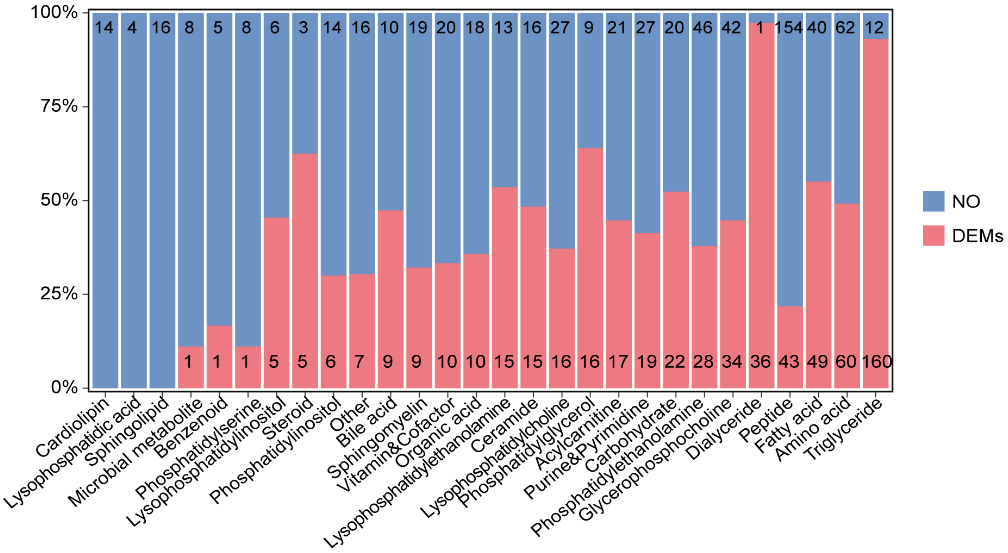

Figure S9. Stacked bar chart of the percentage of differential metabolites in the total detected substances between liver samples from the control group and the *H. pylori* group.

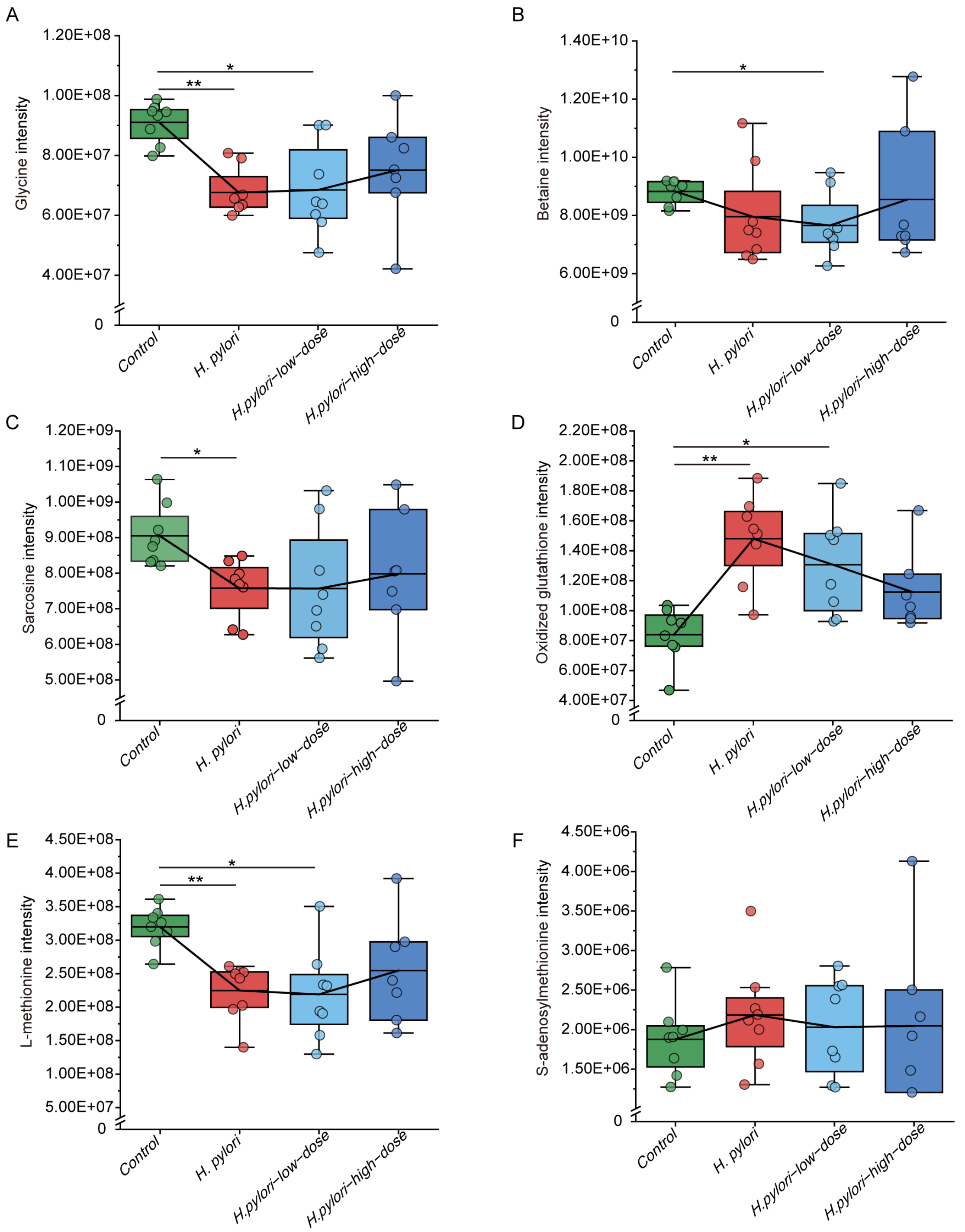

Figure S10. Box plot of the relative concentrations of (A) glycine, (B) betaine, (C) sarcosine, (D) oxidized glutathione, (E) L-methionine, and (F) S-adenosylmethionine in the liver. “*” means $p \leq 0.05$, “**” means $p \leq 0.01$. The *H. pylori*-low-dose and *H. pylori*-high-dose groups received 5 mg/kg/d and 20 mg/kg/d of sulforaphane, respectively.
